# Open-source software to calculate the static sciatic index automatically

**DOI:** 10.1080/17460751.2025.2476390

**Published:** 2025-03-18

**Authors:** Simão Laranjeira, Owein Guillemot-Legris, Gedion Girmahun, James B. Phillips, Rebecca J. Shipley

**Affiliations:** a UCL Department of Mechanical Engineering, London, UK; b Department of Pharmacology, UCL School of Pharmacy, London, UK

**Keywords:** Repair, technology platforms, tools and services, tissue engineering, regeneration

## Abstract

**Background:**

The static sciatic index is commonly used in rat models of nerve crush injury to quantify functional recovery from new therapies under evaluation. However, it is challenging to standardize these measurements across different investigations, and the process is labor intensive.

**Material/methods:**

A new machine learning method was previously developed that performs these measurements automatically and consistently. Here, the approach is tested using two data sets that use different experimental setups, and end-user requirements are evaluated.

**Results:**

The model’s outputs presented a nerve regeneration profile comparable to the manual measurements and outperformed the latter by having a much tighter standard deviation (± 5- ± 10 compared to ± 10 - ± 50).

**Conclusion:**

An inexpensive automatic tool that can perform functional analysis for nerve repair research was developed and tested. The software is available open source to facilitate its dissemination and use in quantifying recovery from peripheral nerve crush injury.

## Plain Language Summary

The rat sciatic nerve crush injury model is a widely used animal model for testing new therapies under development for treating nerve injuries. A commonly used metric to evaluate functional recovery is the static sciatic index (SSI), which is a weighted ratio of distances between the rat’s digits. However, it requires careful measurement of the distance between digits to ensure consistency. This must be done for several rats over numerous time points, becoming a labor-intensive task. High accuracy and consistency in these measurements are important to ensure that the treatment is properly assessed. Here, a new automatic method is proposed that makes use of a machine learning architecture to label the digits and perform the SSI measurements from hundreds of video frames. The detailed computational development of the algorithm has previously been published. Here, an accessible guide for installing and using the model is presented. Additionally, the model is evaluated using two data sets from different setups, illustrating the necessary care required in the data acquisition protocol to ensure the model performs as intended. Furthermore, criteria to avoid common errors are presented. The model is available as an open-source tool, which we hope facilitates its dissemination and uptake to quantitatively assess functional recovery post nerve crush injury.

## 1. Introduction

The rat sciatic nerve crush injury model has become the gold-standard animal model to test novel therapeutic strategies to treat nerve injuries [1]. Usually, one of the animal’s hind legs is injured (and the therapy tested), whilst the other hind leg is used as a control. The sciatic functional index (SFI) and the static sciatic index (SSI) are commonly used to quantify the potential functional recovery that a therapy might induce. These indexes are based on the work by Gutmann and Gutmann [2], which found that the animal’s ability to spread the toes in their hind leg is a reliable metric to track recovery after injury. The SFI uses toe spread and paw length of consecutive steps of a rat walking on a catwalk to quantify functional recovery. By comparison, the SSI is calculated from the toe spread of the animal when it is at rest. Throughout the years, these methods have been optimized [3–6] and shown to be effective at quantifying functional recovery, with the caveat that the toe spread correlates best with recovery from a crush injury. As demonstrated by Valero-Cabré and Navarro [7], when a nerve is severed, neurites might be misdirected as the structures that directed them to the accurate target are not present. Hence, there may be a mismatch between visually-observed nerve regeneration and functional recovery.

**Figure 1.**
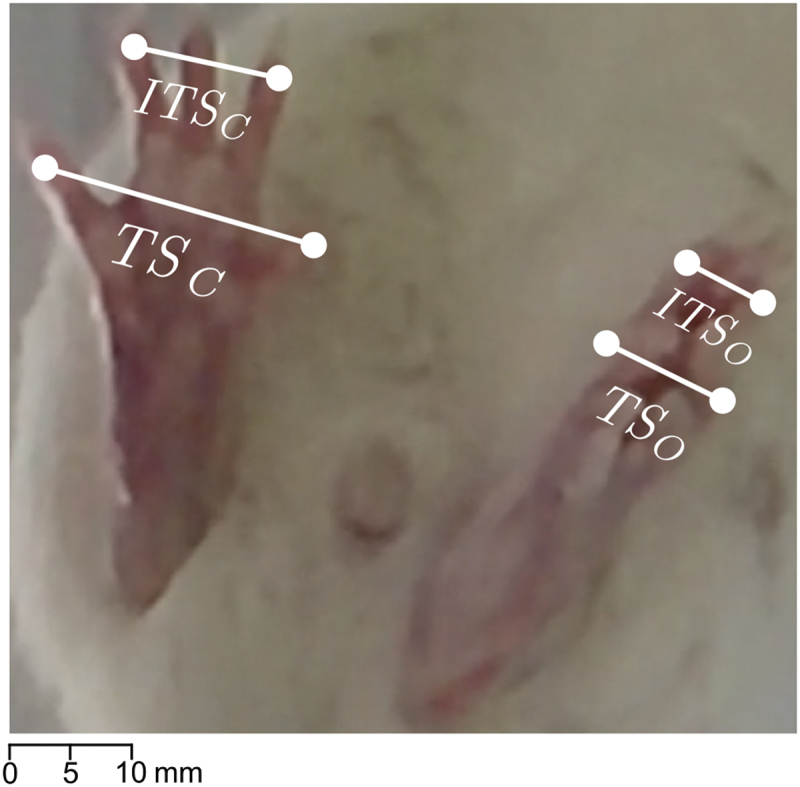
An example of the toe spread measurements used to perform the SSI calculation. For both the operated (o) and the control paws (c), the toe spread (TS) and the intermediate toe spread (ITS) need to be measured.

As demonstrated by Bervar et al. [8], the SSI and SFI measurements are correlated to each other, but the SSI is simpler to calculate and more time and cost-effective. The SSI is calculated as a weighted sum of ratios of digit distances between the operated paw (o) and the control paw (c). Specifically, the intermediate toe spread (ITS) and the toe spread (TS) must be measured for both paws, as depicted in Figure 1.

The intermediate toe spread factor (ITSF) and the toe spread factor (TSF) are then calculated as:

(1) $$\mathrm{TSF}=\frac{TS_{o}-TS_{c}}{TS_{c}},\mathrm{ITSF}=\frac{ITS_{o}-\mathrm{IT}S_{c}}{\mathrm{IT}S_{c}}.$$

Finally, the SSI is calculated as:

(2) $$\mathrm{SSI}=\left(108.44\times \mathrm{TSF}\right)+\left(31.85\times \mathrm{ITSF}\right)-5.49.$$

To measure the distances between the toes, the rats are placed in a transparent box, photographs are taken underneath, and the distance between digits is manually measured using standard software, e.g. ImageJ [9]. This protocol has proven to be labor-intensive as the observer needs to ensure the photos taken are adequate (e.g., the rat is in a position where all the digits are visible) and the distances must be measured from a number of photos (three to five photos), for several time points and rats. Additionally, Monte-Raso et al. [10] demonstrated that when four experts perform the SFI, the measurement varies significantly across the experts (there was no correlation between measurements made by different experts for early time points after the surgery).

Hence, a method that is automatic and consistent is required. There have been several developments in this field. Commercially, several kits perform SSI as well as other measurements but are cost prohibitive. There are also open-source toolboxes that allow researchers to develop their own algorithms to track particular animal features, but require a relatively high level of computational proficiency. Furthermore, there has already been work done to create accessible computational tools that measure the SFI and SSI. However, these require set up consistency and calibration, which require some computational proficiency [11–13]. Here, a tool is provided that is inexpensive, uses open-source software and functions independently of the camera used and set up [14]. Our approach uses a convolution neural network architecture (a U-Net [15]) to segment the digits and then calculate the different toe spreads. The U-Net architecture was chosen because it is efficient at complex segmentation tasks in various contexts when limited data are available [16–18]. The algorithm, through a number of thresholds, is able to detect the direction of the rat and label its paw digits. From this, the algorithm can calculate hundreds of SSI values and uses tested thresholds to remove outliers. In most cases, in a video with 5,000 frames, approximately 20% of those frames are used by the model. Full details on the algorithm development have already been published [14].

In this paper, the performance of the model is evaluated by applying it to two experiments with different experimental setups. Each setup involves placing a rat inside a transparent box on a stand, placing a camera underneath the box, and capturing videos (each 1.5 min long, encompassing 5,000 frames per video). The rat’s regeneration is then quantified by performing the SSI for a number of frames. The setups differ in terms of the box and camera used. Additionally, for one of the setups, the automatic SSI calculations are contrasted against measurements performed manually for between 3 and 5 frames.

**Figure 2.**
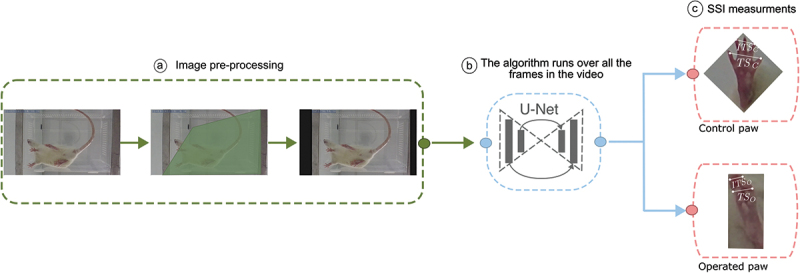
Diagram that describes the three stages of the new method proposed. When the code starts running the user is prompted to crop the area where the rat will walk within, in order to ensure elements around box do not introduce error (I). After all the videos are cropped the labeling process starts in the background (II). For every video, the algorithm identifies the left and right paw and performs the measurements of the TS and the ITS for both paws.

Here, we focus on the algorithm application, which consists of a three-stage process (see Figure 2). First of all, an app window prompts the user to select the source folder for the videos (Figure 2(a)). For each video, cropping must be performed to ensure the model can only see the box where the rat is located (as surrounding elements with similar shapes or hues can introduce noise). After cropping is applied to all the files, the algorithm will run over all the files, labeling the digits, identifying the right from the left paw, calculating from the labels the $TS_{o}$, $TS_{c}$, $\mathrm{IT}S_{o}$ and $TS_{c}$. From these, using Equation 1 the TST and ITSF are calculated and finally hundreds of SSI values, using Equation 2, are produced (Figure 2(b)). All measurements performed and values calculated are saved in an Excel spreadsheet for each individual video (Figure 2(c)).

The work here presented is structured by, firstly, presenting a step-by-step guide to installing the software in different operating systems, as well as the protocols for acquiring video data for the rats (section §2). In §3, the efficacy of the algorithms compared to manual methods is presented for two video data sets. Common data acquisition errors and their implications are discussed in section §4 and the model’s performance is critiqued, the current method’s limitations are presented and adaptions of the model to align with different lab setups are discussed. Finally, all the model’s achievements and future directions for this research are explained in section §5.

## 2. Methods

A novel method has been devised to perform the SSI measurements and released as open-source software [14]. In this section, the two data sets used to test the model are described in §2.1, as well as the procedure used to calculate the SSI by an observer. Then, in section §2.2, the protocol to install and use the software is presented.

### 2.1. Data collection

Four animals from two experiments that implemented the crush sciatic nerve repair model using Wistar rats were chosen. Animal procedures were performed in accordance with the UK Animals (Scientific Procedures) Act (1986) and the European Communities Council Directives (86/609/EEC) and approved by the UCL Animal Welfare and Ethics Review Board (AWERB). The type of repair comprised in wrapping the crushed nerve with a loaded drug delivery device. The drugs tested and their efficacy in promoting nerve regeneration are beyond the scope of this study. In addition, animal selection criteria were based on identifying cases that illustrate the potential of the model and common errors.

**Figure 3.**
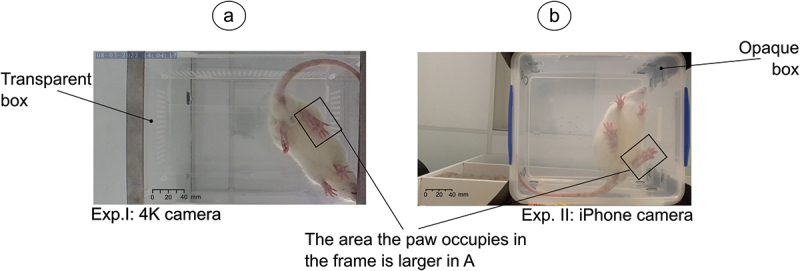
The two setups tested. For exp. I (a) The rats were placed in a transparent box and a 4K camera was used. The setup for exp. II (b), the box is opaque and an iPhone camera is used. The rat is closer to the camera in (a), resulting in the paws having a better resolution compared to (b).

The two experiments vary in terms of the number of data points as well as the setup used to collect the videos. The first (Exp. I) comprises in performing an SSI measurement before surgery (Day 0) and then a data point is collected every 7 days for 12 weeks. The videos were acquired using a 4K camera (Akaso V50 X), and the rats were placed in a fully transparent acrylic box. For the second experiment (Exp. II), a pre-surgery data point is collected and then 6–8 data points are collected until day 28 after the surgery. The setup here consisted of an ‘iPhone’ 8 camera to collect the videos and the rats were placed in an opaque plastic box. For Exp. II, the box was also placed at a larger distance from the camera, which results in the rat’s paws having lower resolution. Figure 3 exemplifies the differences between the two setups. For each experiment, four rats were videoed for 1.5 min corresponding to, approximately, 4,000 frames for Exp. I and 1,800 for Exp. II.

To compare against the current standard methods, manual measurements were also calculated for Exp. I (this is a highly time-consuming task, so we reserved performing this for one experiment only). This was achieved by selecting five frames from each video in which the rat’s paws were clearly visible and then, using Matlab, the digits were labeled manually using the ‘imageLabeler’ app, and the distance between the centroids of the labels was calculated. These distances between digits were then used to calculate the SSI value for each frame, resulting in a mean and standard deviation for each video.

The two experiments each include four rats, chosen specifically to demonstrate a range of the model’s performance. The algorithm is evaluated according to the following metrics: (i) the number of frames in each video the algorithm can automatically label, (ii) the standard deviation of the SSI measurement for a whole video, (iii) the time taken to label a single video and (iv) comparing the SSI measurements calculated manually and via the algorithm (for Exp. I).

### 2.2. Installing and using the open-source software

The software can be found at the GitHub page for this project (https://github.com/Laranjeira-S/SSIapp.git) and the repository downloaded. The software is built using open-source software written in the programming language Python. In addition, to ensure that there are no incompatibilities between toolboxes necessary to run the algorithm, the toolbox manager Anaconda (Anaconda Software Distribution. *Conda*. Version 2–2.4.0, Anaconda, Nov. 2016) needs to be installed. The instructions to install the different software and packages to run the algorithm depend on the operating system, as follows:

#### 2.2.1. Mac OS and Linux

The user can install the software through the terminal, moving to the repository’s directory, and then running the following line: ‘source install_SSIapp.sh.’ This shell script will install a lighter version of the software anaconda called ‘miniconda’ and all the packages that are required to run the algorithm.

#### 2.2.2. Windows

The user must install anaconda from the developer’s website. Then, the necessary conda environment needs to be imported using the command: ‘conda env create -f SSIapp.yml.’

**Figure 4.**
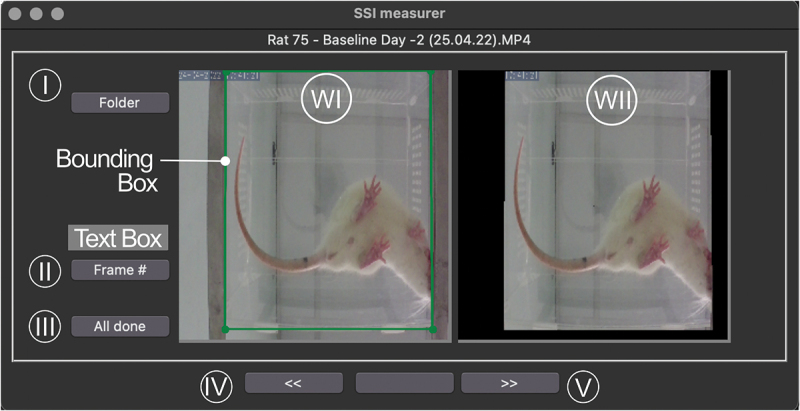
The front-end window of the software app. The user can choose the folder where the videos are located by pressing the ‘Folder’ button (I). A frame from the video will appear in window WI. By clicking in WI the user can place a bounding box to eliminate redundant portions of the frame – the resulting image will appear in window WII. The frame number can be updated via the ‘Frame #’ button (II), and the resulting video can be viewed by pressing the forward arrow (V) (and reviewed via the backwards button (IV)). When satisfied, the user can press the ‘All done’ button (III) and the algorithm architecture shown in Figure 1 runs in the background.

After the last command, a window with the cropping app will appear, and its possible to pre-process the videos can be initiated (see Figure 4). The app consists of two windows, a text box, and five buttons. The user needs to press the ‘Folder’ button (I) and a directory window will open. The user needs to locate the ‘Folder’ where the videos are located. Currently, the algorithm only works with .’mp4’ files. As will be discussed later, changes in video definition impact the model’s performance, and the model was trained with frames from .’mp4’ videos. Hence, its top performance is with this file format.

After the Folder is located, a video frame will appear in the window on the left (WI). If the frame needs cropping to eliminate background structures which are not relevant, a frame number can be inputted in the text box, and the frame will appear after pressing the ‘Frame #’ button (II). Then, cropping is performed by clicking on the frame four times and placing an edge of a polygon each time to trace a connected shape. After the fourth time, a cropped frame will appear in the window on the right (WII). If the cropping is deemed acceptable, the user can move on to the next video by pressing the left arrow (V) at the bottom of the app window. This process is followed for all the videos. The outcomes can be reviewed by moving back using the backward arrow (IV) and amended at any time. If they are all acceptable, the user then needs to press the ‘All done’ button (III), and the algorithm (for image processing, identifying digits, and calculating SSI metrics) will start running in the background. To update any of these options, the algorithm should be stopped and re-run (and all previous options are automatically saved).

The software automatically runs the algorithms for image analysis and calculates the SSI measurements, which are saved in an ‘Output’ folder in the main directory. The latter takes the form of Excel spreadsheets with the same name as the videos, with all the measurements of $TS_{o}$, $\mathrm{IT}S_{o}$, $TS_{c}$ and $\mathrm{IT}S_{c}$ saved, as well as the corresponding TSF, ITSF and SSI, which are calculated based on a random sample of measurements.

## 3. Results

For each animal chosen from the two experiments, all the videos for the time points collected are labeled by the model. For each time point, the mean and standard deviation of the SSI values are calculated from measurements of hundreds of frames.

**Figure 5.**
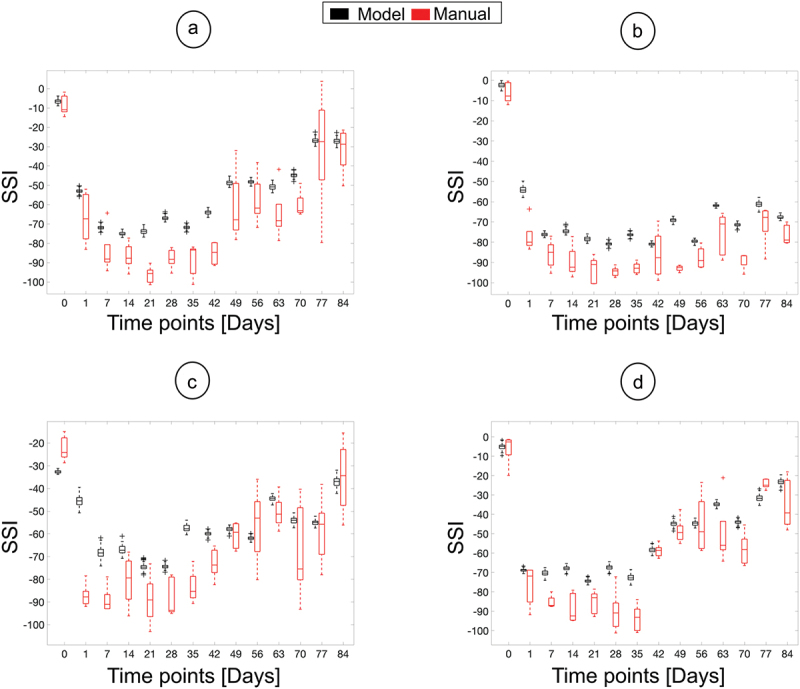
SSI calculations performed manually (red) and by the model (black) for four different animals from Exp. I. The distribution of measurement’s made manually and by the model are plotted as box plots, where the box encompasses the 25th to the 75th quartile of the distribution, the line in the box is the median, the whiskers capture the remaining distribution and the crosses are the outliers.

For Exp. I, SSI measurements were also calculated manually, as presented in Figure 5. The model and manual measurements can be seen to have similar trends over time. Given that the model calculations are based on hundreds of values, the distribution of SSI values for each timepoint is tighter, and therefore outliers can be more readily detected and removed. Resulting in SSI values with a tighter standard deviation (±5 – ±10) when compared to manual measurements (±10 – ±50). Essentially, this indicates a more confident prediction of the SSI values using the model-based approach. However, in Figure 5(b,c), the first time point indicates a big mismatch between the two sets of measurements that will be explained subsequently.

**Figure 6.**
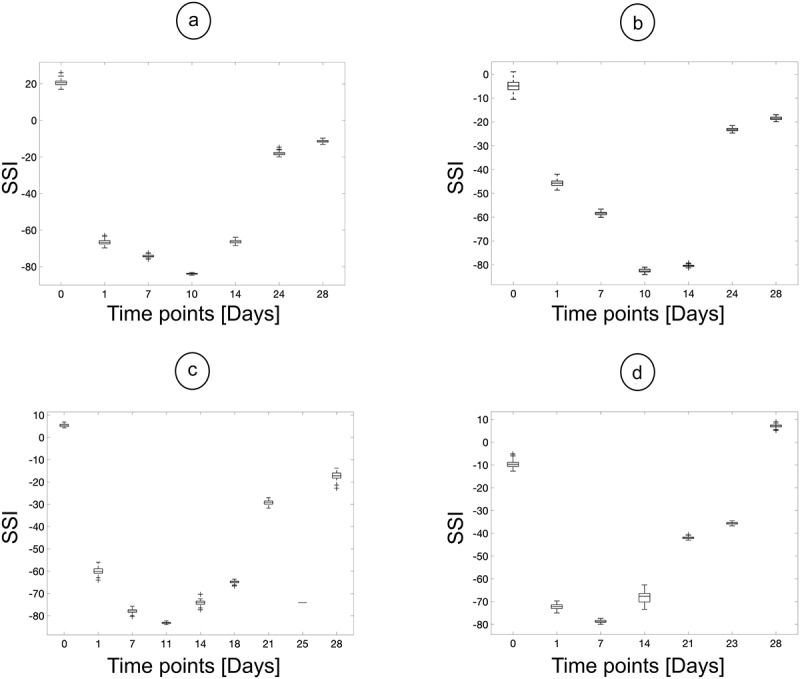
Labelling performed by the model for four different animals from Exp. II, where the distribution of measurements are plotted as box plots. Each box encompasses the 25th to the 75th quartile of the distribution, the line in the box is the median, the whiskers capture the remaining distribution and the crosses are the outliers.

For Exp. II, only the model was used to label the videos for all the time points, with subsequent measurements shown in Figure 6. Here, a similar profile to the evolution of the SSI over time is predicted, as in Figure 5, i.e., the SSI is between −10 and 10 before the surgery is performed, followed by a fall to an SSI value between −60 and −80 and, finally, if there is recovery, the SSI goes back to between −20 and + 10. Additionally, similar standard deviation values to Figure 5 were calculated, between ±5 – ±10. The expected profile is clear in Figure 6(a,d). In Figure 6(b), however, the calculated SSI index decreases initially, reaching a low plateau. Furthermore, in Figure 6(c), one time point (day 25) does not match the expected regeneration profile.

## 4. Discussion

We have described the installation and implementation of the software for automated SSI measurement in any operating system, as well as the comparison of the results against manual techniques. The automated method calculates SSI measurements consistently, and more accurately presenting the animals’ stage of nerve regeneration. Additionally, the new method does not require the purchase of any additional kit and is designed to work in any lab set up. However, the algorithm is dependent on careful acquisition of the videos. In this section, the reasons behind these dependencies is explained as well as possible strategies to ameliorate any errors. Through this process, it is hoped to ensure that the experience of users with the new method is stable, without the need for particular cameras and lighting. Specifically, three typical sources of error are discussed next.

**Figure 7.**
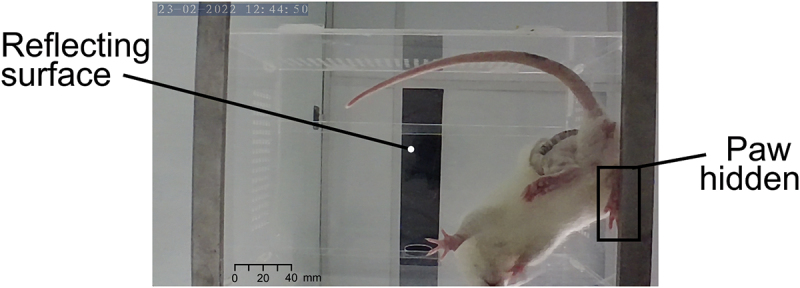
Example frame that demonstrates two features that should be taken into consideration when capturing a video to be labeled by the model: 1. A reflecting surface in the background; care should be taken to ensure the background is homogeneous and 2. one of the animal’s paws is not in full view; the user should ensure that both paws are in view throughout the video.

The first source of error is exemplified by the discrepancy between the manual and model’s SSI measurements in the first data point in Figure 5(b,c). The disparity is because the animals tend to be fidgety when placed in a new environment. This is evident when looking at the manual measurements in Figure 5(a), where the measured SSI values spread is 30 points, and the model’s value is approximately −50, which is close to the top value measured manually. To avoid this error, it is proposed that the animals be allowed to acclimatize in the box before data collection, as seen in Figure 5(d), where the animal was placed twice in the box before the surgery. Then, by day 2 the animal was calm, and the automated SSI measurement was more reliable and comparable with the manual measurements. By comparison, the analogous disparity seen in Figure 5(c) was due to a reflective surface within the frame that introduces noise, and the rat’s paws are not visible in the whole of the video (as summarized in Figure 7).

The case illustrated in Figure 7 presents that there should be extra care taken to ensure there are no confounding features in the bounded area the algorithm can see. This also indicates that the model may have to be recalibrated when there are drastic background changes. Additionally, this might be necessary when different rat species are used as currently the model has only been tested with white coated animals. If this is the case, a folder on the GitHub page provides all the codes necessary to augment the data and re-fit the model.

**Figure 8.**
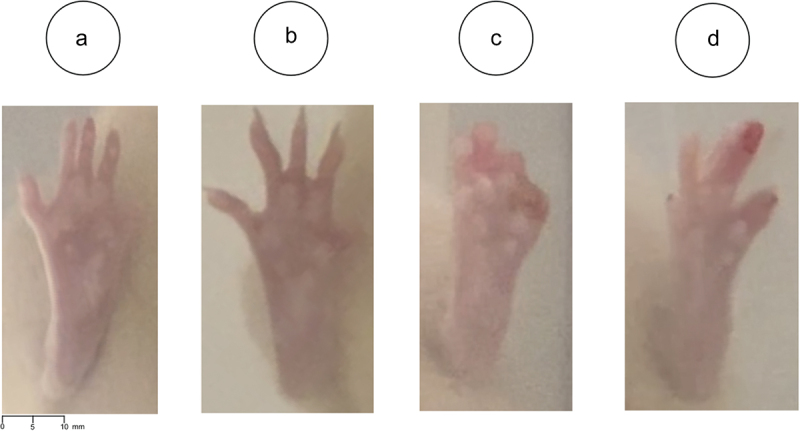
The paw positions that resulted in erroneous measurements from the model in Figure 6. Figure 8(a,b) present the control paw for day 1 and 10 in Figure 6, exemplifying why there is a large difference between those two time points. Figure 8(c,d) show the operated paw for day 21 and 25 from Figure 6(c), explaining why there is a big drop from those two consecutive time points.

The second case can be observed in Figure 6(b) for Exp. II, where the initial time points post-surgery have a larger SSI than may be anticipated. In these experiments, an effort was made to start filming when the rats were stationary. The error here is because the rat’s control paw stays in a position where the digits are not fully extended, resulting in a larger SSI value. In Figure 8(a,b), the control paws for day 1 and day 10 in Figure 6(b) are presented. Similarly, the erroneous measurement for day 25 in Figure 6(c) is due to the placement of the operated (non-control) paw throughout the video. The difference in the placement of the operated paw between days 21 and 25 can be seen in Figure 8(c,d). From these examples, it is clear that unusual positioning of the paws can introduce measurement artifacts. It is then proposed that the model be run concurrently as the videos are collected – this way, amendments can be made to correct any problems due to the paw placement of the rats in real time.

Finally, the third case involves the choice of the camera to perform the measurements. Exp. I used a 4K camera (frame rate 60 fps) whilst Exp. I used an iPhone camera (30 fps). To ensure the model works correctly, between 4,000 and 5,000 frames should be captured; therefore, with a standard camera, around 3 min of film should be collected for each time point. For this number of frames, the algorithm takes approximately 40 min to run over a single video. In terms of resolution, both cameras are comparable, with 1080 × 1920 pixels per frame; however, for the set-up for Exp. II, the distance between the rat and the camera is larger, resulting in the number of pixels a paw occupies being smaller, reducing the resolution of a fully extended paw by 50%. Furthermore, in Exp. II the rats are placed in a box that is opaque, which introduces noise. This results in fewer frames being labeled by the model and therefore being more prone to errors.

All these differences between the two apparatuses resulted in marked differences in measurement calculations when experiments were scaled up to hundreds of videos. For an experiment that use setup of Exp. II and consisting of 260 videos, the model was not able to label an acceptable number of frames (>50) in, approximately, 5% of frames (and in one case not a single frame was labeled). Therefore, it is recommended to capture videos with the highest resolution possible and place the camera as close as possible to the rats, ensuring that the whole area they might move within is covered.

From this analysis, it is demonstrated that the model is able to achieve the desired consistency when data are collected with care, ensuring that:

- the setup uses a box with a fully transparent bottom panel and that the background is homogeneous,
- the camera has the highest resolution possible and, regardless of the camera, around 4,000–5,000 frames per video are collected,
- the rat, throughout the video, has both paws visible and does not stay in a position that is misrepresentative of its stage of regeneration.

## 5. Conclusion

This work contributes to the emerging literature of automating routine animal tracking or measuring animal features repeatedly, specifically those which are time-consuming and labor intensive. While existing tools can perform SSI measurements accurately, they have been found to be cost prohibitive or require technical expertise, as well as strict control over environmental factors such as lighting. The software tool implemented here addresses these challenges, offering a reliable software solution that eliminates the need for specialized equipment or controlled lab conditions.

To demonstrate the potential of our software tool, a user guide is provided using two separate experimental data tests (from testing a drug to promote nerve regeneration post crush injury). The results show that the tool captures the same nerve regeneration profile as the manual measurements with a tighter standard deviation. Hence, the tool developed addresses a concern with the SSI metric regarding consistency of the measurement. Manual methods are not only labor-intensive but also limit the number of frames that can be processed. In contrast, our tool enables the analysis of between 100 and 1,000 measurements per data point, vastly increasing throughput.

Existing computational alternatives require controlled environmental variables to function properly. Conversely, our framework accommodates environmental noise and only requires that the user constrain the relevant area in the video for labeling. With this simple adjustment, our algorithm delivers accurate SSI predictions regardless of the camera used.

Whilst our software tool overcomes many of the limitations associated with SSI measurements, careful data collection is still essential. We have discussed many of these common problems and our solutions. Finally, our model was trained/validated/tested with rats with a white coat; the model may need re-calibration for animals with different colored coats/skin (for example, an albino rat). In summary, many of the protocols used in the field of nerve crush injury repair can be automated by making use of advancements in machine learning. Here, a fully developed tool is demonstrated that can be downloaded and implemented to automate SSI calculations in rat models of nerve crush injury repair, reducing the time burden for experimental researchers and improving consistency of the measurements. With this paper, it is hoped that the software (and encoded model) will reach members of the community who will benefit from such an approach.

## Article highlights

When new therapies for nerve repair are tested *in vivo*, their effect is quantified based on the amount of nerve growth induced and the observed functional recovery.

- The rat sciatic nerve crush model is commonly used to test novel therapies for nerve injury repair. In this model, one paw of the rat is used to test the therapy whilst the other is used as control.
- Functional recovery using this model is commonly quantified using the static sciatic index (SSI), which is based on a correlation between the state of nerve regeneration and the ability of the rat to spread its digits.

It is challenging to ensure consistency in the metrics used to quantify functional recovery both over time and between observers.

- The SSI is calculated as a weighted ratio between the toe spread of the test and control paw, measured at different time points to capture the full nerve regeneration profile. Performing this measurement has been found to be labor intensive.
- Additionally, ensuring consistency of the SSI measurement is difficult due to the influence of errors in manual measurement.

An automatic method that performs a functional recovery metric is proposed that is adaptable to any lab set up and is easy to use.

- A tool that performs the measurement automatically using a convolution neural network architecture is demonstrated. This tool is the first to present an automatic method to perform the SSI that does not require additional kit or controlled environmental variables (e.g. lighting).
- To mitigate environmental error, a GUI was created to allow the user to restrict the area of operation of the algorithm to only where the animal is.

This software tool achieves a more consistent measurement than when performing SSI measurements manually.

- The tool performs hundreds to thousands of measurements, presenting similar regeneration profile to manual measurements with much tighter standard deviation.
- However, there are still cases where the tool underperforms due to problems during data acquisition; approaches for avoiding these errors are identified and evaluated.

The impact of new therapeutic approaches for nerve injury repair must be quantified accurately and systematically; this tool provides an automated method for achieving this, benchmarked against the current gold standard manual approaches.

- This work contributes to the body of literature that seeks to automate experimental techniques that require the tracking and measurement of animal features.

This software tool provides an automated tool for calculating SSI measurements that is more accurate and consistent than manual measurement.
